# Variation in uptake of sodium glucose cotransporter 2 inhibitors and glucagon-like peptide-1 receptor analogues in adults with type 2 diabetes at high cardiovascular risk

**DOI:** 10.1007/s00228-025-03870-2

**Published:** 2025-06-30

**Authors:** Juliana de Oliveira Costa, Jialing Lin, Tamara Y. Milder, Alys Havard, Jerry R. Greenfield, Richard O. Day, Brendon L. Neuen, Alice A. Gibson, Jedidiah I. Morton, Julian W. Sacre, Sallie-Anne Pearson, Michael O. Falster

**Affiliations:** 1 Medicines Intelligence Research Program, School of Population Health, Faculty of Medicine and Health, University of New South Wales, Room 209, Samuels Building (F25), Sydney, NSW 2052 Australia; 2 Department of Diabetes and Endocrinology, St. Vincent’s Hospital, Sydney, NSW Australia; 3 Department of Clinical Pharmacology and Toxicology, St. Vincent’s Hospital, Sydney, NSW Australia; 4 Clinical Diabetes, Appetite and Metabolism Laboratory, Garvan Institute of Medical Research, Sydney, NSW Australia; 5 School of Clinical Medicine, UNSW Medicine & Health, St Vincent’s Healthcare Clinical Campus, University of New South Wales, Sydney, NSW Australia; 6 National Drug and Alcohol Research Centre, University of New South Wales, Sydney, NSW Australia; 7 The George Institute for Global Health, University of New South Wales, Sydney, NSW Australia; 8 Department of Renal Medicine, Royal North Shore Hospital, Sydney, NSW Australia; 9 Menzies Centre for Health Policy and Economics, School of Public Health, Faculty of Medicine and Health, University of Sydney, Sydney, NSW Australia; 10 Baker Heart and Diabetes Institute, Melbourne, VIC Australia; 11 Centre for Medicine Use and Safety, Faculty of Pharmacy and Pharmaceutical Sciences, Monash University, Melbourne, VIC Australia

**Keywords:** Pharmacoepidemiology, Type 2 diabetes, Sodium -glucose cotransporter 2 inhibitor, Glucagon-like peptide-1 receptor agonists

## Abstract

**Purpose:**

We quantified variation in the uptake of sodium-glucose co-transporter 2 inhibitors (SGLT2i) and glucagon-like peptide-1 receptor analogues (GLP-1RA) across sociodemographic, behavioural and clinical characteristics of people with type 2 diabetes (T2D) at high cardiovascular risk.

**Methods:**

We used the 45 and Up Study survey data (2018–2020) linked to dispensing and service claims for 10,171 people with T2D (56% male, median age of 72 years, median diabetes duration of 11 years). We calculated the prevalence of GLP-1RA and SGLT2i use within 1 year and used logistic regressions to assess associations with each participant characteristic.

**Results:**

We found that 2270 (22.3%) people with T2D used SGLT2i and 679 (6.7%) used GLP-1RA. Use of these medicines was higher in people diagnosed with diabetes for a longer period, a high number of comorbidities and survey year, decreased in older people, and varied by sex. After adjusting for these factors, utilisation of these medicines was lower among people who consume alcohol (versus non-drinkers) and higher among those with overweight or obesity. SGLT2i use was also higher in people who were less physically active or had established cardiovascular disease and lower in people with anxiety or depression. GLP-1RA use was higher among people with poorer health and lower in people born outside Australia/New Zealand.

**Conclusion:**

Prevalent use of SGLT2i and GLP-1RA was suboptimal and varied across clinical characteristics and behavioural risk factors. While some variation reflects complexities in prescribing for this older population, there remains opportunity for optimised prescribing within this high-risk population.

**Supplementary Information:**

The online version contains supplementary material available at 10.1007/s00228-025-03870-2.

## Introduction

In the last decade, we have learnt of the cardiorenal benefits of sodium-glucose cotransporter 2 inhibitors (SGLT2i) and glucagon-like peptide-1 receptor agonists (GLP-1RA) in people with type 2 diabetes (T2D). These medicines have shifted the focus of T2D management to early therapy intensification to reduce risk of cardiovascular disease (CVD), cardiovascular events and chronic kidney disease, irrespective of improvements in glycemia [1, 2]. SGLT2i and GLP1-RA are now recommended for use in all people with T2D with atherosclerotic CVD or at high risk of CVD, heart failure or chronic kidney disease, irrespective of glycated haemoglobin (HbA1c) or metformin use [3].

Despite their clinical benefits, SGLT2i and GLP-1RA are being underutilised among people with T2D [4–7]. Identifying populations with suboptimal use of these medicines is key to informing promotion strategies and ensuring optimal uptake — particularly among high-risk individuals who would benefit the most from treatment. For example, older populations with established CVD for which SGLT2i or GLP1-RA medicines have multiple benefits in reducing both the risk of CVD events and chronic kidney disease while reducing other risk factors such as body fat mass, blood pressure and improving lipid profiles [1]. Studies have found uptake differences by sex [5, 6], socioeconomic status [5–7] and geographic remoteness [6, 8]. However, there are many barriers to optimal medicine use, including other sociodemographic, behavioural and clinical factors influencing prescribing and medicine use [9, 10]- for example, clinicians might be hesitant to prescribe for people with increased risk of harms (e.g. alcohol users [11], people with comorbidities[12]). There is limited understanding of how SGLT2i or GLP-1RA use varies across these characteristics, because most prior studies examining the uptake of these medicines have used clinical registries or claims-based datasets which lack information on detailed personal socioeconomic, behavioural and lifestyle factors [6, 7].

Therefore, we leveraged survey data linked to dispensing and service claims data to estimate the prevalence of uptake of SGLT2i and GLP-1RA across a broad range of demographic, socioeconomic, lifestyle and clinical characteristics of people living with T2D at high risk of CVD.

## Methods

### Setting and data source

New South Wales (NSW) is Australia’s most populous state, with approximately 8 million population. The Sax Institute’s 45 and Up Study is a population-based cohort study of NSW residents aged 45 years and over. Participants were randomly sampled from Australia’s universal healthcare system (Services Australia Medicare enrolment database), with an oversampling of people aged 80 + years and residents of rural and remote areas. A total of 267,357 people participated in the study, responding to a baseline survey (2005–2009) and two follow-up surveys (2012–2015 and 2018–2020) and provided consent for their data to be used for research. This corresponded to approximately 11% of NSW population aged 45 years and over (response rate: 19%). Survey data was linked to national dispensing claims from the Pharmaceutical Benefits Scheme (PBS) and to service claims from the Medicare Benefits Schedule (MBS) supplied by Services Australia. Further details of the 45 and Up Study, including participant recruitment, consent and data collection, are described elsewhere [13, 14].

### Medicines of interest

Within Australia’s universal healthcare system, citizens and permanent residents are entitled to subsidised medicines through the PBS. PBS data captures information on PBS-listed medicines dispensed in community pharmacies, private hospitals and on discharge from public hospitals in most states (NSW excluded) [15]. In this study, we examined receipts of SGTL2i and GLP-1RA that are available via the PBS. SGLT2i currently PBS-listed including dapagliflozin, empagliflozin and ertugliflozin and GLP-1RA PBS-listed include exenatide, dulaglutide and semaglutide. During the study period, SGLT2i were subsidised on the PBS for the treatment of T2D if a patient had a glycated haemoglobin (HbA1c) measurement of > 7% (53 mmol/mol) despite treatment with metformin. GLP-1 RA were subsidised on the PBS for the treatment of T2D as add-on therapy to metformin if a patient had a HbA1c > 7% (53 mmol/mol) and had a contraindication to or was intolerant of a sulfonylurea (Supplementary Tables 1–2) [16, 17].

### Study sample

**Fig. 1:**
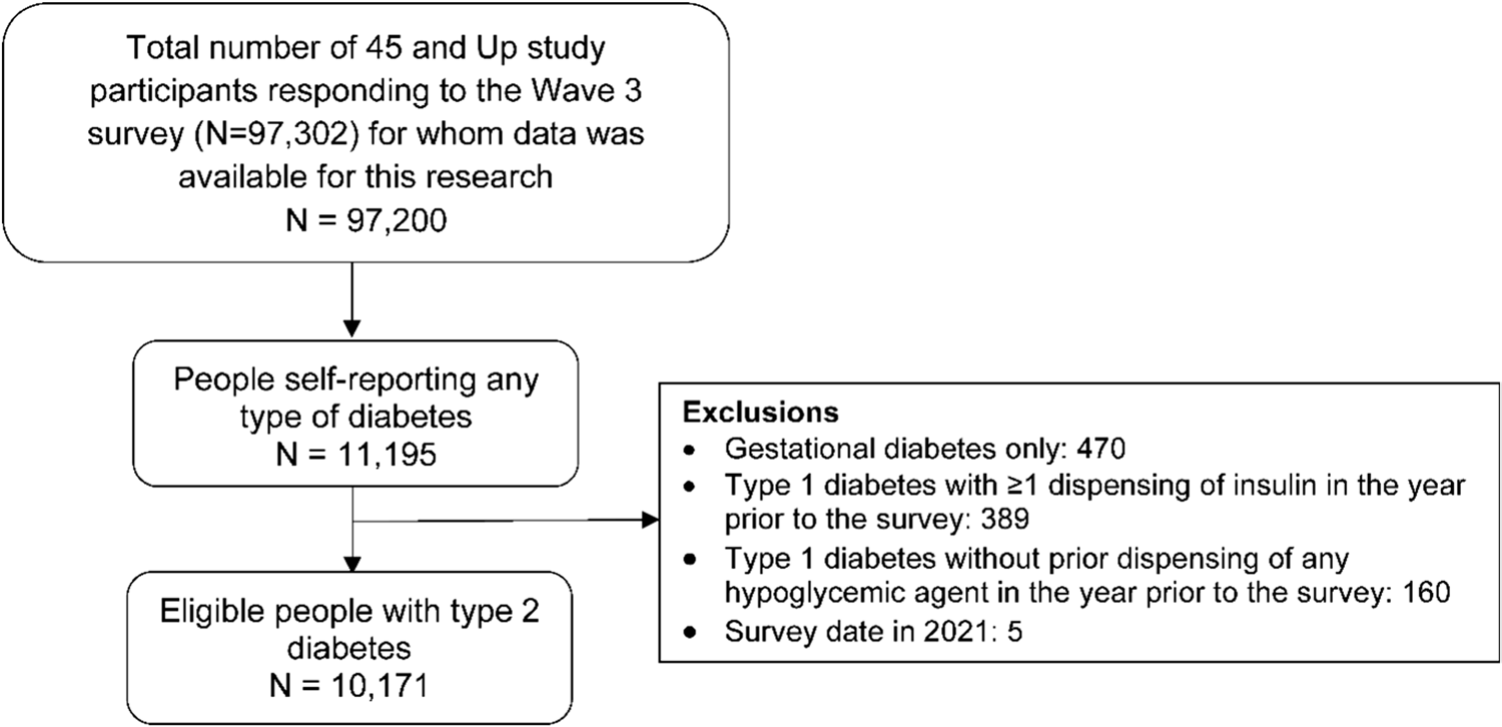
Diagram of study population selection

Of the 97,302 respondents of the 45 and Up Study Wave 3 survey, data from 97,200 were available for this research. Using survey responses and algorithms to ascertain T2D [4, 18, 19], we identified 11,195 people self-reporting diabetes (Has a doctor ever told you that you have diabetes? Answer options: type 1 diabetes (T1D), T2D or unknown, gestational diabetes or not informed), of whom 9900 self-reported having T2D. We then examined PBS dispensing of oral anti-hyperglycaemic agents (OHA) and insulins [4] (Supplementary Table 2) to further identify people with T2D who may have mistakenly self-reported having T1D. We defined T2D among people self-reporting T1D if people had been dispensed OHA but not insulin in the year before the survey (Fig. 1 and Supplementary Fig. 1). Finally, we identified 10,171 people with T2D.

### Study design

We used a cross-sectional study design (Supplementary Fig. 2) to estimate annual rates of prevalent use of medicines among people with T2D according to survey year (2018–2020).

### Outcome measures

We considered people receiving the medicines if they had a dispensing of SGLT2i or GLP-1RA any time from the survey date to 365 days. We presented annual prevalence by demographic, socioeconomic, behavioural, clinical and medicine-related characteristics.

### Covariates

Covariates included survey variables from the second follow-up survey (2018–2020) and baseline survey and variables derived from the administrative datasets (Supplementary Tables 2, 3, 4 and 5). We classified covariates according to the following participant characteristics: sociodemographic (e.g. sex, age group, country of birth, language spoken at home, highest education qualification, region of residence, socioeconomic status), behavioural (e.g. alcohol drinking status, smoking status, physical activity status [defined as moderate to vigorous physical activity (MVPA) [20]]), clinical (e.g. diabetes duration, number of comorbidities [based on medicines dispensed in the prior year [21]], CVD risk [based on age, smoking status, high blood pressure and high cholesterol [22]], established CVD based on self-report, procedures performed and medicines dispensed in the prior year [22], heart failure, depression or anxiety, overweight or obese) and medicine-related (e.g. other anti-hyperglycaemic agent dispensed in the prior year).

### Statistical analysis

We measured use of SGLT2i and GLP-1RA separately, with people dispensed medicines from both classes contributing to both analyses. We conducted descriptive analyses of included participants and characteristics of those dispensed SGLT2i and GLP-1RA by sociodemographic, behavioural, clinical and medicine-related characteristics using proportions for categorical variables and median values and interquartile range (IQR) for quantitative variables.

We stratified annual prevalence by interested covariates and presented as proportions of selected subgroups. For example, we calculated annual prevalence of GLP-1RA use among females as the number of females receiving GLP-1RA by the total number of females with T2D and presented as percentages. We then performed a series of multivariable logistic regressions to test associations of uptake of SGLT2i and GLP-1RA for each participant characteristic. Given a causal framework to inform model selection in this topic is not available and many sociodemographic and clinical variables may be correlated, we fit separate models for each variable adjusted by key factors influencing utilisation. First models were adjusted by age, sex and survey year (Stage 1). Second models were further adjusted for clinical complexity, using participant’s self-reported duration of diabetes and number of comorbidities (Rx-Risk Comorbidity Index[21], measured on medicines dispensed in the year prior to survey) (Stage 2). Therefore, we fit two separate adjusted models for each variable of interest. Missing values were excluded from the regression models.

## Results

### Cohort characteristics

Our study cohort was older (median age 72 years [IQR: 66, 78]), with a large proportion aged 65–74 years (41.6%). Over half were male, approximately three-quarters were born in Australia or New Zealand and less than 10% spoke languages other than English at home. Participants were highly educated (57% with higher education degree), and half lived in major cities.

Approximately half self-reported having T2D for 11 years or more. Most people had three or more comorbidities (86%), had a high CVD risk profile (96%), had overweight or obesity (76%) and one third had established CVD. Moreover, over half self-reported drinking every week, and over a third did not perform 150 min or more of moderate to vigorous physical activity per week.

Most people had a PBS-dispensing of metformin in the year prior to the survey (71.5%), with variable rates of use of other anti-hyperglycaemic agents. More details are shown in Table 1.

**Table 1:** Characteristics of 45 and Up Study participants of the most recent follow-up survey with type 2 diabetes, NSW (2018–2020)

Characteristics	*N* (%)
All	10,171 (100.0)
Survey year	
2018	2211 (21.7)
2019	3380 (33.2)
2020	4580 (45.0)
*Sociodemographic*	
Sex (Male)	5648 (55.5)
Age, years, median (IQR)	72 (66, 78)
Age group (years)	
55–64	1951 (19.2)
65–74	4234 (41.6)
75–84	3142 (30.9)
85 or more	844 (8.3)
Country of birth (Not Australia/New Zealand)^a^	2239 (22.0)
Language spoken at home (not English)^a^	902 (8.9)
Highest education qualification^a^	
University or higher	2404 (23.6)
Certificate/diploma/trade	3481 (34.2)
High school	942 (9.3)
School certificate	2136 (21.0)
No school or qualification	1096 (10.8)
Unknown	112 (1.1)
Quintile of Relative Socioeconomic Disadvantage	
1 st quintile—most disadvantaged	2280 (22.4)
2nd quintile	2290 (22.5)
3rd quintile	1802 (17.7)
4th quintile	1630 (16.0)
5th quintile—least disadvantaged	1728 (17.0)
Unknown	441 (4.3)
Region of residence^b^	
Major cities	5123 (50.4)
Inner regional	3625 (35.6)
Outer regional	1001 (9.8)
Remote/very remote	79 (0.8)
Unknown	343 (3.4)
*Behavioural*	
Alcohol use	
Non-drinker	2814 (27.7)
Ex-drinker	288 (2.8)
Drinker (1–6 drinks per week)	2759 (27.1)
Drinker (7 or more drinks per week)	2556 (25.1)
Unknown	1754 (17.3)
Smoking status	
Never smoked	5461 (53.7)
Ex-smoker	4238 (41.7)
Current smoker	332 (3.3)
Unknown	140 (1.4)
Moderate or vigorous physical activity in the prior week (150 min or more)	
Yes	4614 (45.4)
No	4310 (42.4)
Unknown	1247 (12.3)
*Clinical*	
Diabetes duration, years median (IQR)	11 (6, 19)
Diabetes duration, years	
0–5	2221 (21.8)
6–10	2014 (19.8)
11–15	1734 (17.1)
16 or more	3212 (31.6)
Unknown	990 (9.7)
Number of comorbidities^c^, median (IQR)	5 (3, 7)
Number of comorbidities^c^	
0–2	1329 (13.1)
3–5	4719 (46.4)
6 or more	4032 (39.6)
Unknown	91 (0.9)
High blood pressure ever (Yes)	6395 (62.9)
Established cardiovascular disease (Yes)	3021 (29.7)
Heart failure: cardiac failure, weak heart, enlarged heart (Yes)	1208 (11.9)
Overweight or obese (BMI ≥ 25)	
Yes	7679 (75.5)
No	1666 (16.4)
Unknown	826 (8.1)
Depression or anxiety (Yes)	2318 (22.8)
Overall health assessment	
Excellent/Very good/Good	7151 (70.3)
Fair/poor	2843 (28.0)
Unknown	177 (1.7)
*Medicine-related*	
Glucose-lowering agents dispensed in the year prior to the survey (people may contribute to multiple groups)	
None	1985 (19.5)
Metformin	7270 (71.5)
Sulfonylureas	2151 (21.2)
Insulin	1609 (15.8)
DPP4i	2422 (23.8)
GLP-1RA	513 (5.0)
SGLT2i	1889 (18.6)
Other	84 (0.8)

*IQR* interquartile range, *BMI* body mass index, *DPP4i* dipeptidyl peptidase-4 inhibitors, *GLP-1RA* glucagon-like peptide-1 receptor agonists, *SGLT2i* sodium-glucose cotransporter 2 inhibitors

^a^From baseline survey

^b^Derived from postcode at recruitment

^c^Based on medicines dispensed in the prior year

### Annual prevalence of SGLT2i use

A total of 2270 (22,3%) people with T2D were dispensed any SGLT2i within 1 year from the survey, most of which (75.2%) also had an SGLT2i dispensing in the year prior. SGLT2i utilisation was higher among males than females (25.4% vs 18.4%, *p* value < 0.001) and lower with increasing age (from 30.2% in people aged 55–64 years to 5.8% among those aged ≥ 85 years, *p* value < 0.001). Use was higher in more recent survey years (from 19.4% in 2018 to 24.0% in 2020, *p* value < 0.001).

**Fig. 2:**
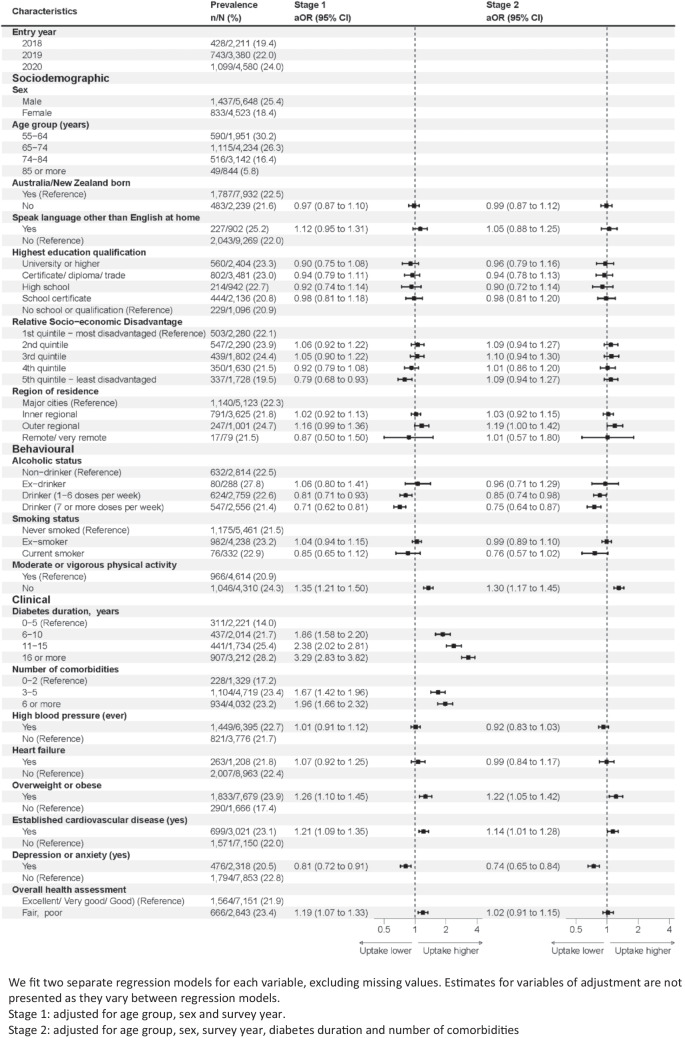
Annual prevalence of SGLT2i use and adjusted odds ratios (aOR) by participant characteristics (2018–2021)

After adjusting for these key sociodemographic characteristics (age, sex and survey year; Fig. 2, stage 1), SGLT2i use was higher in people living in most disadvantaged areas than least disadvantaged areas. Regarding behavioural characteristics, SGLT2i use was lower in alcohol drinkers than non-drinkers and less physically active people than physically active people. Regarding clinical characteristics, SGLT2i use was higher with longer diabetes duration and more comorbidities. Use was also higher in people who are overweight or obese and people who had established CVD and self-reported fair/poor health. Use was lower among people who self-reported anxiety or depression.

After further adjusting for key markers of clinical complexity (number of comorbidities and diabetes duration; Fig. 2, stage 2), there was no statistically significant difference in utilisation of SGLT2i by socioeconomic status. However, use remained lower in alcohol drinkers, less physically active people and people who self-reported anxiety or depression. It remained higher in people who were overweight or obese and people who had established CVD.

### Annual prevalence of GLP-1RA use

A total of 679 (6.7%) people with T2D were dispensed GLP-1RA within 1 year from the survey, most of whom (*n* = 425) also had a GLP-1RA dispensing in the year prior. GLP-1RA utilisation was higher among females than males (7.8% vs 5.8%, *p* value < 0.001) and was lower with increasing age (from 9.6% in people aged 55–64 years to 1.8% among those aged ≥ 85 years, *p* value < 0.001). Utilisation was higher in more recent survey years (from 5.1% in 2018 to 8.2% in 2020, *p* value < 0.001).

**Fig. 3:**
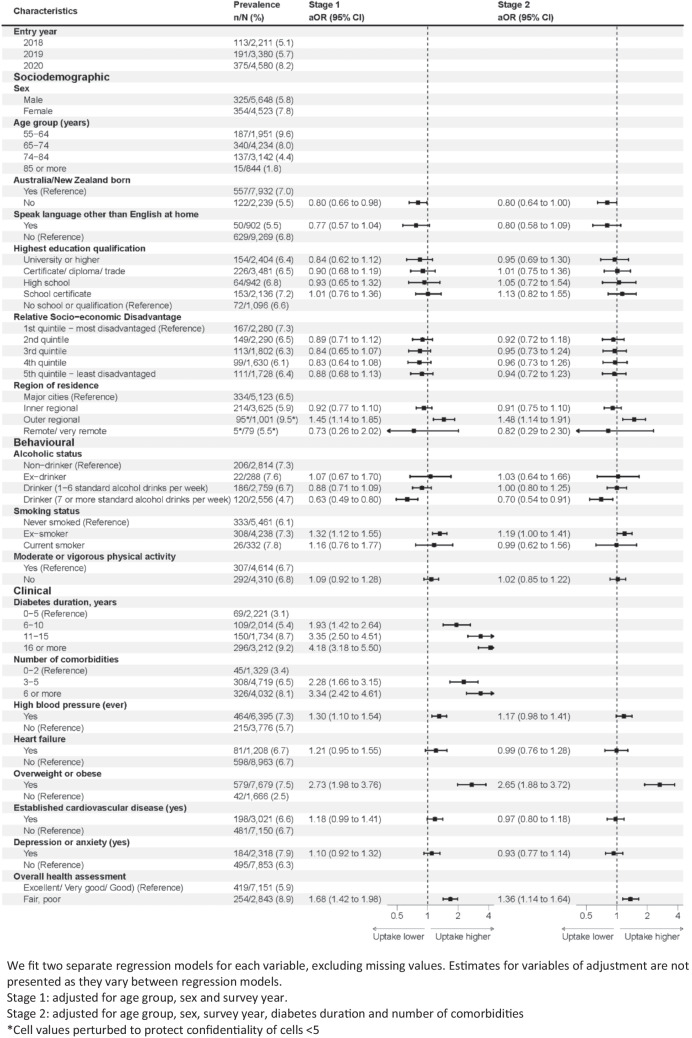
Annual prevalence of GLP-1RA use and adjusted odds ratios (aOR) by participant characteristics (2018–2021)

After adjusting for these key sociodemographic characteristics (Fig. 3, stage 1), use of GLP-1RA was lower in people born outside of Australia or New Zealand (than people born within these countries), as well as in people living in outer regional areas than people living in major cities. Regarding behavioural characteristics, use of GLP-1RA was lower in drinkers with higher levels of alcohol consumption and higher among ex-smokers than never smokers. Regarding clinical characteristics, use of GLP-1RA was higher with longer diabetes duration and more comorbidities. It was also higher in people who self-reported having high blood pressure, people who were overweight or obese or had reported fair or poor health.

After further adjusting for key markers of clinical complexity (Fig. 3, stage 2), there were few statistically significant associations. Use of GLP-1RA was marginally lower in people born in countries other than Australia or New Zealand. Use was also higher in people living in outer regional areas than people living in major cities, people who were overweight/obese and people with fair/poor health. Use was lower in people with higher levels of alcohol consumption.

## Discussion

We found uptake of SGLT2i and GLP-1RA was suboptimal among older adults living with T2D in NSW, with less than one quarter (22.3%) or people using an SGLT2i, and 1 in 15 (6.7%) people using a GLP-1RA. Given the high risk of cardiovascular events often experienced in older populations, continued investigation into health system barriers for prescribing and access is needed to ensure optimal use of these medicines in people who would benefit the most. Encouragingly, we found utilisation rates increased in more recent years and did not observe variation in uptake by sociodemographic factors. While we also found higher levels of use among people with clinical factors which aligned with need for therapy—such as people with more comorbidities, cardiovascular risk factors or behaviours (such as insufficient physical activity) and longer diabetes duration—the use of these medicines was still low within these population groups.

### Suboptimal use in the Australian context

Multiple studies have demonstrated increasing use of SGLT2i and GLP-1RA among people with T2D in Australia and internationally, particularly after the cardio-renal benefits of these medicines have been increasingly evident in the last decade.[4, 5, 23–26]. However, given the potential benefits, overall utilisation remains low, with less than one in four people living with T2D receiving these medicines [SGLT2i (~ 20%) and GLP-1RA (~ 6%) as of 2019] [5, 24]. This is consistent with other population-level studies finding low use among older populations [8].

With regard to the clinical appropriateness of the use of these medicines, a Swedish study based on national registry data estimated around 50–80% of people with T2D would be eligible to receive either SGLT2i or GLP1-RA based on 2019 clinical guideline recommendations [27]. While our study population was primarily older, and we could not assess eligibility to receive these medicines, the high levels of cardiovascular risk factors and CVD indicate that a large proportion of our study population could benefit from receiving SGLT2i or GLP-1RA—and hence this was a potential missed opportunity. During the observation period of our study, these medicines were recommended as preferred add-on therapies to prevent further risk of progression and cardiovascular and renal complication in both international (2019) and local guidelines (2020) [27, 28].

During the study period, the PBS required people to meet specific criteria for subsidy of SGLT2i or GLP1-RA, such as use in combination with metformin and having a HbA1c ≥ 7% despite present treatment. Data from a clinical audit in Australia found two in three patients with T2D had an HbA1c ≥ 7% and 73% were using metformin. Thus, approximately 70% of people with T2D would be eligible for second-line therapy according to PBS reimbursement criteria required during the observation period of our study [29]. Taken altogether, use of SGLT2i and GLP-1RA was suboptimal even if we accounted for clinical and PBS reimbursement indications.

### Use according to behavioural and clinical factors

Despite the low use across the board, we found behavioural and clinical variation in the use of SGLT2i and GLP1-RA aligned with evidence-based practice, with targeted use in specific populations most likely to benefit from these medicines. For example, we observed higher utilisation among people who were overweight or obese, as well as people with low physical activity, aligning with the weight loss benefits of these medicines, particularly for GLP1-RA [5, 25]. We also found lower utilisation among alcohol drinkers, which might be due to concern about an increased risk of the rare serious side effect of SGLT2i diabetic ketoacidosis [30] and a potentially higher risk of pancreatitis with GLP1-RA. Interestingly, GLP-1 RA is being explored as a potential treatment for alcohol use disorders [31].

We also found higher utilisation of SGLT2is in people with established CVD, diverging from previous research in Australia, suggesting a non-selective uptake by the presence of CVD [5, 26]. This might reflect the fact we analysed more recent data and that this is a rapidly evolving area. There is increasing prescriber awareness of the use of SGLT2i for preventing heart failure hospitalisations and kidney disease reflecting recent guideline recommendations from diabetes and cardiac societies [32, 33]. Similarly, the higher association with number of comorbidities and diabetes duration may reflect increased prescribing in high-risk populations for such secondary prevention. However, we also found lower rates of SGLT2i use among people reporting anxiety or depression. The reason for this difference is unclear.

### Use according to sociodemographic factors

Similar to other studies [34, 35], we found lower use of SGLT2i and GLP1-RA therapies with increasing age which may reflect prescriber concern about side effects in the older age population. This is despite increased prevalence of cardiorenal disease in this population and other treatments for T2D having potentially serious risks, for example sulfonylureas and hypoglycaemia.

Although we did not find statistically significant differences in utilisation across a range of other sociodemographic factors, we did observe some differences indicative of potential disparities. For example, we found lower use of GLP-1RA in people born outside of Australia and New Zealand. A previous study in Australia reported general practitioners in training to prescribe GLP-1RA or SGLT2i less often to people from non-English-speaking background [36], possibly due to communication challenges between prescribers and their clients. While we did not find this association when assessing language spoken at home, this association was borderline significant (potentially due to the small subgroup size with < 10% of our sample speaking languages other than English).

Lower utilisation of SGLT2i and GLP1-RA in remote areas has been reported in Australia when these medicines were first introduced in PBS [6]. Although we did not find utilisation decreased with increasing remoteness, similar to a recent study using 45 and Up data [5], the lowest utilisation rates were observed in people with T2D living in remote areas. We also had limited power to analyse this group, given the small proportion of people living in remote areas. However, we found people living in outer regional areas were more likely to receive GLP-1RA compared to people living in major cities even after adjusting for age, sex, number of comorbidities, and diabetes duration, with a marginal effect for SGLT2i. These findings may reflect an increasing dissemination of evidence of the benefits of these medicines among clinicians practising in those areas. It is well known that regional areas have lower availability of specialists, and general practitioners in outer regions may have an expanded scope of practice compared with those in major cities, possibly being more confident in prescribing add-on therapies for T2D management than their major city counterparts [12]. This result may also reflect a higher prevalence of specific cardiovascular conditions and risk factors that underpin the indication for prescription of these medicines in regional areas compared to major cities. It may also relate to outreach programs rolled out to increase the uptake of these medicines in these areas [37]—with a recent population-level study finding highly localised patterns of increased GLP-1RA use in regional areas [8].

### Implications for clinical practice and policy

The generally low level of use of SGLTT2i and GLP-1RA suggests that addressing broader health system barriers may help improve uptake of these medicines[38]—such as prescriber education initiatives, as well as medicine subsidisation. In Australia, several reasons for suboptimal use of newer cardiometabolic medicines have been identified, such as lack of confidence in prescribing SGLT2i [12, 39], under-appreciation of their cardio-renal benefits [12, 39] and concerns with potential adverse effects and prescribing among people with comorbidities [12], which may be common concerns when prescribing amongst older populations. While costs of medicines have not been raised by clinicians as a barrier to prescribing [12], monthly therapy with GLP1-RA and SGLT2i is substantially costlier than older add-on options such as sulfonylureas to general PBS beneficiaries (AUD$30 vs. around $10 in July 2023) [40]. Utilisation of SGLT2i is likely to increase in more recent years, as the PBS glycaemic requirement for subsidised access for the treatment of T2D was lifted in December 2024 to facilitate access among people with high cardiovascular risk [41]. However, for GLP-1RA, there have been more reimbursement restrictions since March 2023, that only those contraindicate or intolerant to SGLT2i are eligible to reimburse GLP-1RA for treating T2D [42].

### Limitations

Our study has several limitations. We used a highly selective cohort of people with T2D participating from the 45 and Up Study. This population is unlikely to be representative of the whole NSW T2D population in terms of age, health status and sociodemographics. While population-level extrapolation of utilisation rates should be performed with caution, internal relative risk estimates from this data have been found to be comparable with those from population health surveys [43]. We used self-reported information and dispensing data to identify the T2D population. While consistent with previous studies identifying people with T2D [4, 18], there is potential for misclassification due to recall bias and private dispensings not captured from PBS data. Similarly, there may be misclassification in other self-reported variables. We also lacked data on key indications and contraindications, such as HbA1c (a key biomarker of diabetes control and indicator for eligibility for SGLT2i and GLP1-RA via the PBS) and further potential factors influencing uptake (e.g. prescriber preferences). This means we could not assess appropriateness of prescribing and may be underestimating the prevalence of indicated use within our study population. Despite those limitations, the availability of survey data containing other key sociodemographic, clinical and behavioural characteristics linked to PBS data enabled a deeper understanding of a broader range of factors related to the utilisation of these critical medicines. During the study period, there were shortages of SGLT2i and GLP1-RA, although all shortages were classified as non-critical and possibly had a limited impact on our analysis [23]. Further research from different health systems and population groups is needed to confirm the generalisability of these findings and to unpack the role of specific associations where there may be common factors influencing medicine use (e.g. obesity and physical exercise; prescriber preferences).

In conclusion, the uptake of SGLT2i and GLP-1RA in a cohort of older and comorbid people living with T2D was suboptimal. Despite variation in the uptake of these medicine classes by sociodemographic, behavioural and clinical characteristics of people with T2D, further systemic interventions to promote prescribing within high-risk populations may be needed to increase utilisation of these highly efficacious medicines.

## Supplementary Information

Below is the link to the electronic supplementary material.

Supplementary file1 (DOCX 193 KB)

## Data Availability

The data were provided by the Sax Institute. Access to the data by other individuals or authorities is not permitted without the express permission of the approving human research ethics committees and data custodians.
