# Supplementary material for: Variation in uptake of sodium glucose cotransporter 2 inhibitors and glucagon-like peptide-1 receptor analogues in adults with type 2 diabetes at high cardiovascular risk
Source: Eur J Clin Pharmacol. 2025 Jun 30;81(9):1315–27. doi: 10.1007/s00228-025-03870-2 (PMC12398476; doi:10.1007/s00228-025-03870-2)
Supplement: Supplementary file 1 — Supplementary file1 (DOCX 193 KB) [file 228_2025_3870_MOESM1_ESM.docx]

**Sociodemographic, behavioural and clinical variation in uptake of sodium glucose co-transporter 2 inhibitors (SGLT2i) and glucagon-like peptide-1 receptor analogues (GLP-1RA) in adults with type 2 diabetes at high risk of cardiovascular disease**

**Supplementary Appendix**

**Contents**

[**Study measurements** 2](#_Toc197587273)

[**Supplementary Table 1:** PBS-listed glucagon-like peptide-1 receptor analogues (GLP-1RA) and sodium-glucose co-transporter 2 inhibitors (SGLT2i) available during the study period (up to December 2021) 2](#_Toc197587274)

[**Supplementary Table 2:** World Health Organization Anatomical Therapeutic Chemical Classification (ATC) Codes for identifying other medicines used in the year prior to the survey date 3](#_Toc197587275)

[**Supplementary Table 3:** Variables selected from survey data 4](#_Toc197587276)

[**Supplementary Table 4:** Algorithm to identify people with established cardiovascular disease using administrative data and self-report. The condition is attributed if a participant meets any of the criteria across and within categories 6](#_Toc197587277)

[**Supplementary Table 5:** Algorithm to identify people at high-risk of CVD. CVD risk factor is attributed if a participant meets all criteria on a row based on all surveys available. 7](#_Toc197587278)

[**Supplementary Figure 1.** Algorithm to define the study population. 8](#_Toc197587279)

[**Supplementary Figure 2.** Study design diagram 9](#_Toc197587280)

# **Study measurements**

# **Supplementary Table 1:** PBS-listed glucagon-like peptide-1 receptor analogues (GLP-1RA) and sodium-glucose co-transporter 2 inhibitors (SGLT2i) available during the study period (up to December 2021)

| Generic name | First PBS-listed | Mode of use* | PBS Item Code |
| --- | --- | --- | --- |
| *GLP-1RA* |  |  |  |
| Exenatide | Aug 2010 | Twice daily injection | 03423E, 03424F, 10888C |
| Dulaglutide | Jun 2018 | Once-a-week injection | 11364D |
| Semaglutide | Jul 2020 | Once-a-week injection | 12075M, 12080T |
| *SGLT2i* | |  |  |
| Canaglifozin | Dec 2013 – Aug 2015 | Once-daily oral tablet | 02987F, 02873F |
| Dapagliflozin | Dec 2013 | Once-daily oral tablet | 10011X, 11291G |
| Empagliflozin | Jan 2015 | Once-daily oral tablet | 10202Y, 10206E, 11281R, 11314L |
| Ertugliflozin | Dec 2018 | Once-daily oral tablet | 11570Y, 11571B, 11577H, 11585R |
| Dapagliflozin + metformin | Oct 2015 | Once-daily oral tablet | 10510E, 10515K, 10516L, 11270E,11300R, 11313K |
| Empagliflozin + metformin | Mar 2016 | Once-daily oral tablet | 10626G, 10627H, 10633P, 10639Y, 10640B, 10649L, 10650M, 10677Y |
| Saxagliptin + dapagliflozin | Apr 2018 | Once-daily oral tablet | 11286B, 11305B |
| Empagliflozin + linagliptin | Apr 2018 | Once-daily oral tablet | 11298P, 11303X, 11310G, 11269D |
| Ertugliflozin + metformin | Dec 2018 | Twice daily oral tablet | 11562M, 11563N, 11564P, 1568W  11569X, 11575F, 11581M, 11584Q |
| Ertugliflozin + sitagliptin | Dec 2018 | Once-daily oral tablet | 11561L, 11578J, 11579K, 11583P |

*According to the Australian Handbook of Medicines

# **Supplementary Table 2:** World Health Organization Anatomical Therapeutic Chemical Classification (ATC) Codes for identifying other medicines used in the year prior to the survey date

| Medicine class | ATC code |
| --- | --- |
| *Glucose-lowering agents* | A10AA – A10BX |
| Insulin | A10AA – A10AE |
| Sodium-glucose co-transporter 2 inhibitors and combinations | A10BK01, A10BD15, A10BD21, A10BK03, A10BD19, A10BD20, A10BK04, A10BD23, A10BD24, A10BX11 |
| Glucagon-like peptide 1 receptor analogues | A10BJ01, A10BJ05, A10BJ06 |
| Metformin | A10BA02 |
| Metformin combinations (SGLT2i) | A10BD15, A10BD20, A10BD23 |
| Metformin combinations (other classes) | A10BD02, A10BD03, A10BD07, A10BD08, A10BD10, A10BD11, A10BD13 |
| Sulfonylureas | A10BB, A10BD02 |
| Dipeptidyl peptidase-4 inhibitors | A10BH, A10BD07, A10BD08, A10BD10, A10BD11, A10BD13,  A10BD19, A10BD21, A10BD24 |
| Thiazolidinediones | A10BG, A10BD03 |
| Other (Thiazolidinediones, acarbose) | A10BG, A10BD03, A10BF |
| *Cardiovascular medicines* |  |
| Antihypertensives | C03A, C03BA04, C03BA11, C08, C09, C10BX |
| Loop diuretics | C03B |
| Lipid lowering agents | C10 |
| Anticoagulants | B01A |
| Antiplatelets | B01AC |
| Beta-blockers | C07 |
| Loop diuretics | C03C |
| Vasodilators | C01D |
| Potassium-sparing diuretics | C03D, C03E |
| Antiarrhythmics | C01B |
| Glycosides | C01AA |
| Other cardiovascular groups | C01EB17, C02AB, C02AC, C02CA, C03DB, C02DC |

# **Supplementary Table 3:** Variables selected from survey data

| **Question** | **Response options** | **Group** | **Variable name** | **Source** |
| --- | --- | --- | --- | --- |
| Accessibility/Remoteness Index of Australia (2006) ARIA+ mean (derived from postcode at recruitment) |  | Address | ARIA_plus_mean | Wave 3 |
| Socioeconomic status - SEIFA 2006: Index of Relative Socio-economic Disadvantage (derived from postcode at recruitment) |  | Address | SEIFA_2006_IRSD | Wave 3 |
| What is your date of birth? |  | Age and Gender | yearbirth | Baseline |
| What is today's date? |  | Age and Gender | fup2_datentoday | Wave 3 |
| Age (derived from date of birth and date today) | Age | Age and Gender | Age | Wave 3 |
| What is your gender? | Male Female | Age and Gender | Gender | Wave 3 |
| How tall are you without shoes? | Centimetres Feet Inches | Height, Weight and BMI | fup2_height | Wave 3 |
| About how much do you weigh? | Kilograms Stone Pounds | Height, Weight and BMI | fup2_weight | Wave 3 |
| BMI (derived from height and weight) |  | Height, Weight and BMI | BMI | Wave 3 |
| What is the highest qualification you have completed? | No school certificate or other qualifications School or intermediate certificate (or equivalent) Higher school or leaving certificate (or equivalent) Trade/apprenticeship (e.g. hairdresser, chef) Certificate/diploma (e.g. child care, technician) University degree or higher | Qualifications and Employment | highestqual | Baseline |
| In which country were you born? | Australia, UK, Ireland, Italy China, Greece, New Zealand Germany, Lebanon, Philippines Netherlands, Vietnam, Malta, Poland, Other (please specify) | Cultural Background | Country born | Baseline |
| Do you speak a language other than English at home? | Yes No | Cultural Background | otherlanghomeyn | Baseline |
| Have you ever been a regular smoker? | Yes No | Smoking and Alcohol | fup2_smoeverregyn | Wave 3 |
| Are you a regular smoker now? | Yes No | Smoking and Alcohol | fup2_smoregnowyn | Wave 3 |
| About how many alcoholic drinks do you have each week? |  | Smoking and Alcohol | fup2_alcdrinksperweek | Wave 3 |
| If you add up all the time you spent doing each activity LAST WEEK, how much time did you spend ALTOGETHER doing each type of activity? | Walking continuously Vigorous physical activity Moderate physical activity | Physical Activity/Inactivity | fup2_vigourtotalweek fup2_modtotalweek | Wave 3 |
| Has a doctor ever told you that you have: | Heart failure (cardiac failure, weak heart, enlarged heart) (Wave 2 on)  Other heart disease (Wave 2 on) Atrial fibrillation (Wave 2 on) High blood pressure - when not pregnant (F) High blood pressure (M) Stroke Diabetes - type 1 (Wave 3 on) Diabetes - type 2 or unsure (Wave 3 on) Diabetes - gestational (F) (Wave 3 on) Depression Anxiety | Medical Conditions Overall | **Heart failure:**  fup2_evertoldhrtfyes  **Other heart disease:**  fup2_evertoldothhrtyes **Atrial fibrillation:** fup2_evertoldatrfibyes **High blood pressure (men):** fup2_evertoldhighbldyes **High blood pressure (female):** fup2_evertoldhighbldothyes **Stroke:** fup2_evertoldstrokeyes **Type 1 diabetes:** fup2_evertolddiab1yes **Type 2 diabetes:** fup2_evertolddiab2yes **Gestacional diabetes:** fup2_evertolddiabgyes **Depression:** fup2_evertolddepression2yes **Anxiety:** fup2_evertoldanxiety2yes | Wave 3 |
| In the last month have you been treated for: | Heart attack or angina High blood pressure High blood cholesterol | Treatments overall | **Heart attack or angina:** fup2_trthrtattackyes **High blood pressure:** fup2_trhighbldyes **High blood cholesterol:** fup2_trhighcholyes | Wave 3 |
| Have you ever had any of the following operations? Heart or coronary bypass surgery | Yes No | Operations Overall | operationheartyes | Baseline |
| In general, how would you rate your: Overall health | Excellent, Very good, Good Fair, Poor | Self-rated Health | fup2_ratehealth | Wave 3 |
| At present do you consider yourself: | A non-drinker An ex-drinker An occasional drinker A light drinker A social drinker A heavy drinker A binge drinker | Smoking and Alcohol | fup2_alcdrinkertype | Wave 3 |

Reference: Sax Institute. Data and technical information: Access 45 and Up Study questionnaire variables, data books and data dictionaries. Available at: https://www.saxinstitute.org.au/solutions/45-and-up-study/use-the-45-and-up-study/data-and-technical-information/.

# **Supplementary Table 4:** Algorithm to identify people with established cardiovascular disease using administrative data and self-report. The condition is attributed if a participant meets any of the criteria across and within categories

| Established CVD |  |
| --- | --- |
| **Medicare Benefits Schedule (MBS) items indicating** | MBS item number |
| **Ischaemic Heart Disease** |  |
| Transluminal coronary angioplasty | 38300, 38303 |
| Transluminal coronary angioplasty with stenting | 38306 |
| Percutaneous transluminal rotational atherectomy of coronary artery | 38309, 38312, 38315, 38318 |
| Coronary artery bypass | 38496, 38497, 38498, 38500, 38500, 38501, 38503, 38504 |
| Re-operation for reconstruction of occluded coronary artery graft | 38637 |
| Percutaneous transluminal rotational atherectomy | 38309, 38312, 38315, 38318 |
| Open coronary endarterectomy | 38505 |
| Myocardial infarct study | 61310 |
| *Ischaemic stroke* |  |
| Exploration of the carotid artery | 34100 |
| Cartoid endarterectomy | 33500 |
| Embolus removal from artery of neck | 33800 |
| **Pharmaceutical Benefits Scheme dispensings of:** | Anatomical Therapeutic Chemical code |
| Glyceryl trinitrate | C01DA02 |
| Isosorbide dinitrate | C01DA08 |
| Isosorbide mononitrate | C01DA14 |
| Nicorandil | C01DX16 |
| Perhexeline Maleate | C01DX |
| **Self-report** |  |
| Diagnosis of stroke, heart failure or other heart diseases, treatment for heart attack or angina | |
| Operation for heart disease of transient ischaemic attack (from baseline survey) | |

Adapted from:

- Liu Z., *et al*. Using Large-Scale Linkage Data to Evaluate the Effectiveness of a National Educational Program on Antithrombotic Prescribing and Associated Stroke Prevention in Primary Care. Journal of the American Heart Association. 2016 5(10).

# **Supplementary Table 5:** Algorithm to identify people at high-risk of CVD. CVD risk factor is attributed if a participant meets all criteria on a row based on all surveys available.

| Diabetes | Age | Smoker (ever or current) | High BP | High cholesterol |
| --- | --- | --- | --- | --- |
| Yes | ≥60 | - | - | - |
| Yes | 55 - 59 | No | Yes | Yes |
| Yes | 55 - 59 | Yes | - | Yes |
| Yes | 55 - 59 | Yes | Yes | - |
| Yes | 45 - 54 | Yes | Yes | - |
| Yes | 45 - 54 | Yes | - | Yes |

Liu Z., *et al*. Using Large-Scale Linkage Data to Evaluate the Effectiveness of a National Educational Program on Antithrombotic Prescribing and Associated Stroke Prevention in Primary Care. Journal of the American Heart Association. 2016 5(10).


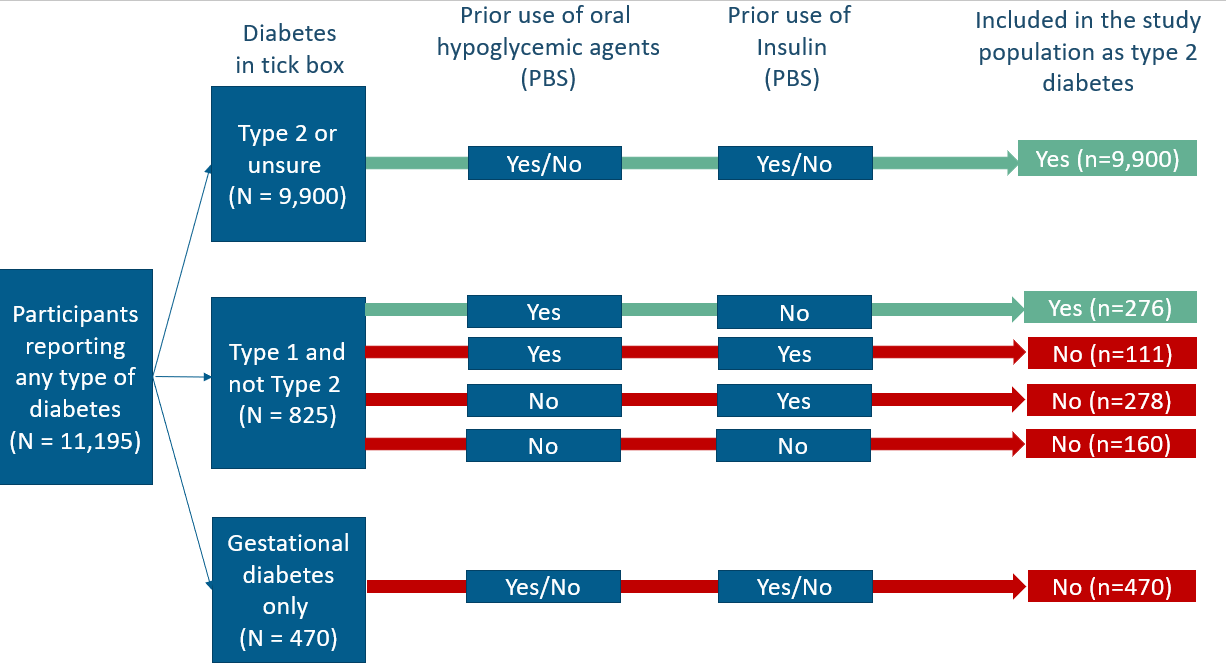


# **Supplementary Figure 1.** Algorithm to define the study population.

PBS: Pharmaceutical Benefits Scheme.

Note: Prior use of oral hypoglycemic agents was defined as having at least one dispensing of antidiabetic medicines (Anatomical Therapeutic Chemical code: A10) other than insulin in the Pharmaceutical Benefits Scheme (PBS) in the year prior to the questionnaire completion. Out of 9,900 people self-reporting type 2 diabetes, 301 also reported type 1 diabetes and 346 also reported gestational diabetes.


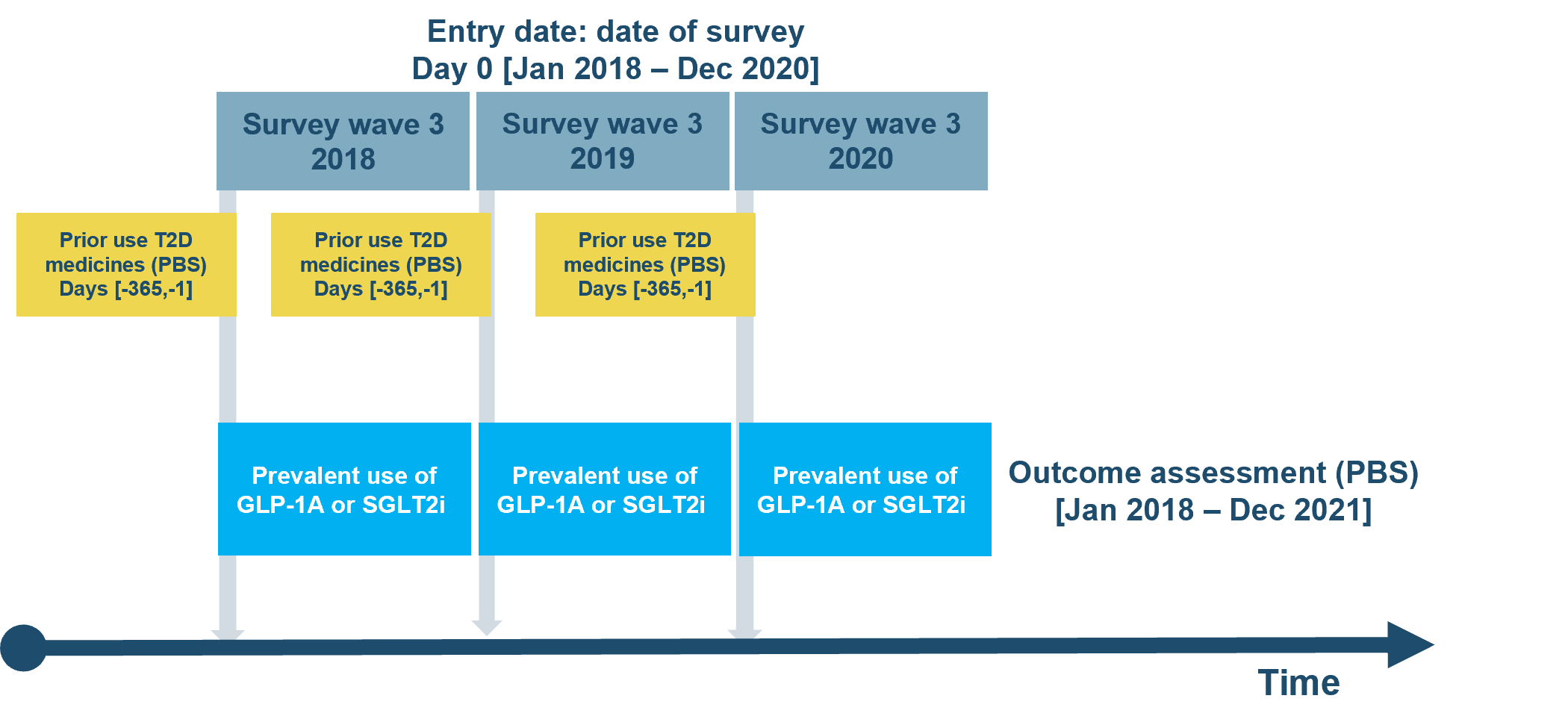


# **Supplementary Figure 2.** Study design diagram

Note: Prior use of type 2 diabetes (T2D) medicines was defined as having at least one dispensing of antidiabetic medicines (Anatomical Therapeutic Chemical code: A10) other than insulin in the Pharmaceutical Benefits Scheme (PBS) in the year prior to the questionnaire completion.
